# CD8 T Cell Sensing of Type I Interferon Impacts Anergy

**DOI:** 10.1002/eji.70192

**Published:** 2026-05-07

**Authors:** Anne S. Haefke, Hanna Gröber, Ioana Sandu, Vanessa Skipness, Nikos Pantouloufos, Fabienne Gräbnitz, Marie‐Elen Tuchel, Nathan Zangger, Dominique Stark, Marvin Kříž, Carmine Cristinzio, Hanspeter Pircher, Roman Spörri, Isaak Quast, Annette Oxenius

**Affiliations:** ^1^ Institute of Microbiology ETH Zurich Zurich Switzerland; ^2^ Max Planck Institute of Immunobiology and Epigenetics Freiburg Germany

**Keywords:** anergy, autoimmunity, CD8 T cells, peripheral tolerance, type I interferon

## Abstract

Peripheral tolerance is indispensable for the maintenance of immune homeostasis, allowing protective immunity while limiting responses to self‐antigens. CD8 T cells activated in the absence of co‐stimulation and pro‐inflammatory cytokines are either deleted, rendered anergic, or actively suppressed. These mechanisms are well established, but the cues determining the mode and depth of peripheral tolerance remain incompletely understood. Here, we identify type I interferon (IFN‐I) signalling in T cells as a key modulator of peripheral tolerance in the absence of infection. In the complete absence of IFN‐I signalling, autoreactive CD8 T cells are rendered anergic, and their expansion, phenotype and function are tightly controlled. Basal levels of IFN‐I are sufficient for self‐reactive CD8 T cells to expand and retain partial effector functions in the absence of viral infections. This is dependent on T cell‐intrinsic IFN‐I sensing and is associated with the generation of a partially anergic, TCF1^+^ CD8 T cell subset that can contribute to a pathogen‐specific immune response. Collectively, our results suggest that elevated basal IFN‐I levels limit anergy induction, providing a potential mechanistic explanation for the association of baseline inflammation with the development of autoimmunity.

## Graphical abstract

Basal IFN‐I acts as a rheostat to fine‐tune CD8 T cell anergy. The presence of IFN‐I induces a less deep anergic state characterised by greater expansion, residual effector function and plasticity. This favours the accumulation of autoreactive T cells that retain the potential to break tolerance rather than undergoing deletion. Created in BioRender (2026). https://BioRender.com/y0syq8s

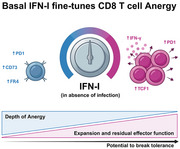

**Abbreviations**
ALT: alanine aminotransferase
DC: dendritic cells
ffu: focus forming units
GP: glycoprotein
IFNAR: interferon a/b receptor
IFN‐I: type I interferon
ISG: IFN‐stimulated gene
LCMV: lymphocytic choriomeningitis virus
MCMV: murine cytomegalovirus
MFI: mean fluorescence intensity
Sca‐1: stem cell antigen‐1
SLE: systemic lupus erythematosus
T1D: type I diabetes
tg: transgenic
Treg: regulatory T cell
wt: wild type

## 1 Introduction

Peripheral tolerance ensures that mature, self‐reactive CD8 T cells that have escaped negative selection in the thymus do not mount effector immune responses against self‐antigens [1, 2]. This is maintained through complementary mechanisms, including active suppression by regulatory T cells (Treg) [3], clonal deletion [4, 5] and the induction of anergy [6, 7, 8]. Anergy is a reversible, unresponsive state that is actively induced by exposure of naïve CD8 T cells to antigen in the absence of co‐stimulation and inflammation [9, 10]. The determining factors governing these outcomes (deletion vs. persistence of anergic CD8 T cells) of peripheral CD8 T cell tolerance remain incompletely resolved. The fate of CD8 T cells is influenced by the strength of their initial activation via the T cell receptor (TCR) (signal 1) [6, 11], the extent of co‐stimulation (signal 2) [9] and inflammation (signal 3) [12, 13]. As such, the priming of a naïve CD8 T cell in the presence of self‐antigen, signal 2 and signal 3 can perturb peripheral tolerance induction and drive autoimmune pathology [14, 15].

Type I interferons (IFN‐Is) are among the main components of inflammation in response to viral infection and shape CD8 T cell expansion, differentiation, and effector function [16, 17, 18]. All IFN‐Is bind to the interferon‐α/β receptor (IFNAR) [19] and trigger JAK‐STAT, insulin receptor substrate/PI‐3‐kinase and MAPK signalling to regulate cell survival, protein translation and RNA degradation and to induce interferon‐stimulated genes (ISGs) [20, 21, 22]. In CD8 T cells, IFN‐I signalling acts in tandem with TCR engagement to promote proliferation and effector differentiation via STAT‐4 [23]. Notably, TCF1, a key regulator of CD8 T cell fate, was found to antagonise terminal differentiation upon chronic TCR stimulation in the presence of IFN‐I, supporting the persistence of progenitor‐like CD8 T cells with proliferative and differentiation potential [24]. Conversely, IFN‐I signalling before TCR stimulation exerts antiproliferative and pro‐apoptotic effects via STAT‐1 [23]. Also, dysregulated IFN‐I production can lower the threshold for T cell activation and compromise tolerance, and elevated IFN‐I levels are associated with increased disease severity of multiple autoimmune diseases, including systemic lupus erythematosus (SLE), Sjögren's syndrome, or type I diabetes (T1D) [25, 26, 27]. Collectively, these findings underscore the crucial role of IFN‐I at the intersection of immune defence and self‐tolerance.

Here, we explore how steady and low levels of IFN‐I shape autoreactive CD8 T cell responses. Specifically, we examine how IFN‐I, sensed by self‐reactive CD8 T cells, influences their deletion or the induction and extent of anergy. Our results show that CD8 T cells primed by self‐antigens in the presence of elevated, but non‐inflammatory, levels of IFN‐I can expand, persist and retain partial effector functions alongside exhibiting features of anergy. Moreover, IFN‐I signalling in the absence of infection promotes the emergence of an anergic stem‐like subset that is marked by TCF1 expression, and has superior potential to expand and regain effector functions upon viral challenge. Taken together, these findings suggest that IFN‐I fine‐tunes peripheral CD8 T cell responses, enabling self‐reactive cells to persist in a functionally restrained yet responsive state and thereby reveal the stringency of self‐tolerance.

## 2 Results

### 2.1 CD8 T Cell Tolerance in MHCI.GP and H8 Hosts Is Differentially Induced

To study tolerance induction to self‐antigens *in vivo*, we used TCR transgenic CD8 T cells specific for amino acids 33–41 derived from the lymphocytic choriomeningitis virus (LCMV) glycoprotein (gp_33‐41_), known as P14 cells [28]. We adoptively transferred P14 cells into two different transgenic recipient lines expressing gp_33‐41_ to study the induction of peripheral tolerance: First, mice expressing a fragment of the LCMV glycoprotein (GP), including the gp_33‐41_ epitope, inserted into the N‐terminus of the H‐2K^b^ heavy chain, known as H8 mice [29]. Second, MHCI.GP mice (also known as GP_WE_ mice), which express the entire LCMV GP under the control of the H‐2K^b^ promoter [30]. In both mouse strains, this results in gp33/H‐2D^b^ presentation on all nucleated cells, which has previously been reported to induce peripheral T cell tolerance of gp_33‐41_ specific CD8 T cells after a period of transient activation [29]. H8 and MHCI.GP strains do not exhibit signs of autoimmunity or pathology [29, 30] as central tolerance remains intact and endogenous CD8 T cells specific for gp_33‐41_ are deleted in the thymus.

**FIGURE 1:**
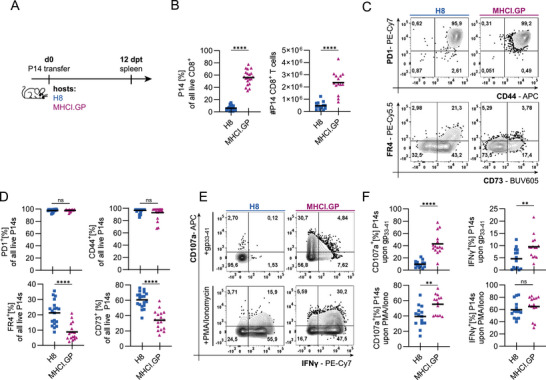
Differential CD8 T cell tolerance induction in MHCI.GP and H8 hosts. (A) Experimental design to investigate peripheral CD8 T cell tolerance. Naïve TCR‐transgenic CD8 T cells (P14 cells) were adoptively transferred into recipients (H8 or MHCI.GP) that ubiquitously express their cognate antigen, and splenocytes were analysed 12 days thereafter. (B) Frequency and number of splenic P14 cells. (C) Representative flow cytometry plots showing PD1, CD44, FR4 and CD73 expression by P14 cells and (D) quantification. (E) Representative flow cytometry plots showing IFN‐γ production and degranulation (CD107a) by P14 cells from H8 or MHCI.GP hosts following 6 h *ex vivo* restimulation with either gp_33‐41_ or PMA/Ionomycin and (F) quantification. (A–F) Data are pooled from three to four independent experiments. (B, D, F) Data are shown as individual biological replicates + mean. *p*‐values were calculated with an unpaired *t*‐test. **p < 0.01, ****p < 0.0001.

Twelve days after adoptive transfer, spleens were isolated, and the phenotype and function of P14 cells were analysed (Figure 1A). P14 cell expansion was minimal in H8 hosts, while P14 cells expanded notably in MHCI.GP hosts (Figure 1B). In both H8 and MHCI.GP hosts, P14 cells expressed high levels of CD44, consistent with activation and antigen encounter, and upregulated the co‐inhibitory receptor PD1. However, P14 cells isolated from MHCI.GP hosts expressed significantly less anergy‐associated markers (FR4, CD73) than P14 cells isolated from H8 hosts (Figure 1C,D). P14 cells from MHCI.GP hosts also retained more effector functions, such as granule exocytosis (indicated by cell surface CD107a expression) and IFN‐γ secretion, upon *in vitro* stimulation with gp_33‐41_ (Figure 1E,F). These differences were less pronounced upon stimulation with PMA/Ionomycin, which bypasses TCR‐proximal signalling [31]. In both H8 and MHCI.GP hosts P14 cell transfer did not translate into clinical symptoms of autoimmunity, as indicated by the absence of weight loss and unchanged serum liver enzymes (alanine aminotransferase, ALT) (Figure S1). The observed differences in expansion, phenotype and function of P14 cells isolated from H8 or MHCI.GP hosts were maintained at least until day 30 post‐adoptive cell transfer (Figure S2).

As TCR downstream signalling pathways remain functional in anergic cells [32], PMA/Ionomycin treatment allows cytokine production despite the proximal signalling block characteristic of T cell anergy (Figure 1E,F). In line, P14 cells derived from both hosts exhibited comparable Ca^2+^ flux upon stimulation with PMA/Ionomycin (Figure S3A), but cells derived from MHCI.GP hosts showed an increased response to stimulation via agonistic antibodies to CD3 and CD28 (Figure S3B). To investigate proximal TCR signalling, we crossed P14 mice with NUR77 reporter mice (*Nr4a1*‐GFP), which express GFP under the control of the *Nr4a1* promoter [33]. We analysed GFP expression by NUR77 reporter P14 cells isolated from H8 and MHCI‐GP hosts. To allow comparison with signalling occurring in response to cognate antigen encounter in the context of an infection, we transferred *Nr4a1*‐GFP P14 cells into non‐transgenic (wt) recipient mice and infected i.v. with 200 focus‐forming units (ffu) LCMV WE, resulting in an acute infection that is cleared within 7 days (Figure S3C). Elevated baseline *Nr4a1*‐GFP expression was observed in anergic P14 cells isolated from H8 and MHCI.GP hosts in comparison to P14 cells isolated from LCMV‐infected wt recipients post‐viral clearance (10 days post‐infection, dpi) (Figure S3D). *In vitro* restimulation with gp_33‐41_‐loaded dendritic cells (DC) or agonistic anti‐CD3/CD28 antibodies resulted in stronger *Nr4a1*‐GFP expression by P14 cells derived from MHCI.GP hosts compared with those from H8 hosts, while both showed deficits when compared with P14 cells from LCMV‐infected recipients (Figure S3E). PMA/Ionomycin stimulation again resulted in less pronounced differences. Taken together, while P14 cells in both hosts acquire features of peripheral tolerance [34, 35, 36, 37, 38], the anergic state induced in MHCI.GP hosts is characterised by increased expansion, partial acquisition of effector functions and reduced expression of molecules associated with anergy.

### 2.2 Anergic CD8 T Cells Maintain Their Phenotype During Persistent Self‐antigen Exposure

**FIGURE 2:**
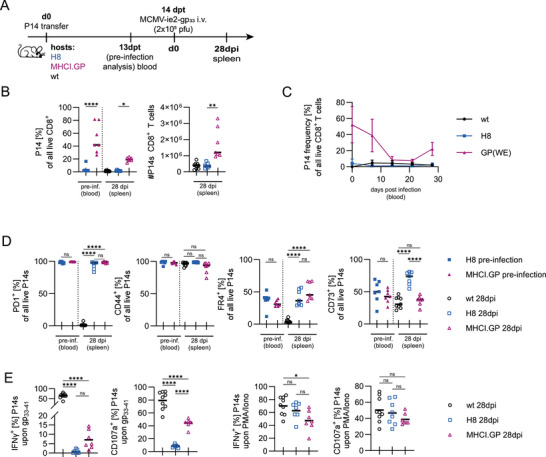
Long‐term stability of CD8 T cell anergy in the presence of continuous self‐antigen exposure. (A) Experimental design. (B) P14 cell frequencies in blood pre‐infection (13 days post cell transfer, one day before MCMV infection) and frequencies and total cell numbers in the spleen 28 dpi. (C) Expansion of P14 cells following MCMV infection, with data lines indicating group mean + SD. (D) Proportion of P14 cells from H8 versus MHCI.GP hosts expressing PD1, CD44, FR4 or CD73. (E) IFN‐γ production and CD107a expression of P14 cells isolated from respective hosts and *ex vivo* restimulation with either gp_33‐41_ or PMA/Ionomycin for 6 h. (A–E) Data are pooled from two independent experiments. Unless stated otherwise, data points indicate individual mice, with lines indicating the data median. *p*‐values were calculated with ANOVA with Tukey's post hoc test. ns = not significant, *p < 0.05, **p < 0.01, ****p < 0.0001.

We next assessed if these differences in anergy influenced the ability of P14 cells to respond to their cognate antigen in the context of an infection while remaining in their self‐antigen‐rich environment. An equal number of naïve P14 cells was adoptively transferred into H8, MHCI.GP, or wt hosts, and 14 days thereafter, mice were infected i.v. with a recombinant murine cytomegalovirus (MCMV) expressing the gp_33‐41_ epitope under the control of the immediate early 2 promoter (MCMV‐ie2‐gp_33‐41_) (Figure 2A), as previously described [39]. The immune control of MCMV‐ie2‐gp_33‐41_ occurs irrespective of the CD8 T cell response to gp_33‐41_, limiting potential effects that a variation in viral titres may have on P14 cells. Upon infection, P14 cells remained more abundant in MHCI.GP hosts than in H8 hosts (Figure 2B) due to both increased expansion and superior maintenance into the memory phase (Figure 2C). P14 cells showed almost no phenotypic changes, with both populations retaining high CD44 and PD1 expression, accompanied by a small increase in the anergy‐associated markers CD73 and FR4 (Figure 2D). Functionally, P14 cells isolated from MHCI.GP and H8 hosts displayed reduced granule exocytosis and IFN‐γ secretion when compared with P14 cells isolated from MCMV‐ie2‐gp_33‐41_‐infected wt hosts. These defects were again less pronounced in MHCI.GP than in H8 hosts and almost absent in response to PMA/Ionomycin stimulation (Figure 2E). Taken together, the unaltered phenotype of self‐reactive CD8 T cells in H8 and MHCI.GP mice after viral infection suggests that the anergic state in a self‐antigen‐rich environment is stably maintained and refractory to inflammation‐induced activation.

### 2.3 Removal From Self‐Antigen Exposure Allows Previously Anergic TCF1^+^ CD8 T Cells to Regain Effector Functions

**FIGURE 3:**
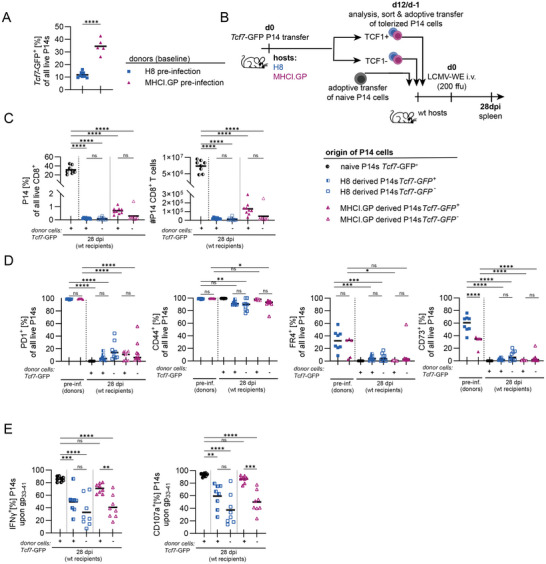
Removal from self‐antigens and tolerising environment enables *Tcf7*‐GFP^+^ CD8 T cells to regain effector function. (A) *Tcf7*‐GFP expression among P14 cells 12 days post‐adoptive transfer. (B) Experimental design for (C–E) to assess the response to infection by autoreactive CD8 T cell subsets from H8 and MHCI.GP mice upon removal from autoantigen and tolerogenic environment. (C) P14 cell frequency and numbers post re‐transfer and LCMV infection (28 dpi). (D) Frequency of PD1, CD44, FR4 and CD73 expression among P14 cells from H8 versus MHCI.GP hosts on the day of re‐transfer and 28 dpi. (E) IFN‐γ production and CD107a expression of P14 cells isolated from respective hosts and *ex vivo* restimulation with either gp_33‐41_ or PMA/Ionomycin for 6 h. (A–E) Data are pooled from two independent experiments. Data points indicate individual mice, with lines indicating the median data. *p*‐values were calculated with ANOVA with Tukey's post hoc test. ns = not significant, *p < 0.05, **p < 0.01, ***p < 0.001, ****p < 0.0001.

Expression of the transcription factor TCF1 by CD8 T cells indicates ‘stemness’, reflected by increased self‐renewal, proliferative capacity and long‐term survival [40]. Among functionally exhausted CD8 T cells, TCF1 marks a progenitor‐like subset which retains some potential to proliferate and differentiate in response to antigen encounter [41]. To test whether a stem‐like population also persists within anergic, tolerant CD8 T cells, we crossed P14 mice with *Tcf7*‐GFP reporter mice [41]. P14 cells isolated from MHCI.GP hosts 12 days post adoptive cell transfer retained more TCF1 expression (reported by *Tcf7*‐GFP) than P14 cells from H8 hosts (Figure 3A). The expression of CD73 and PD1 did not differ between *Tcf7*‐GFP^+^ and *Tcf7*‐GFP^−^ P14 cells within H8 or MHCI.GP hosts, whereas the fraction expressing FR4 was reduced in *Tcf7*‐GFP^−^ P14 cells from MHCI.GP hosts (Figure S4A). To examine whether the depth of the imprinted anergic state is altered upon removal from their self‐antigen‐rich environment, and to investigate potential functional differences in TCF1^+^ and TCF1^−^ cells, we sorted *Tcf7*‐GFP^+^ or *Tcf7*‐GFP^−^ P14 cell subsets from H8 and MHCI.GP hosts (Figure S4B) and adoptively transferred them into separate naïve wt recipients. As a control, naïve P14 cells (uniformly expressing *Tcf7*‐GFP) were isolated and transferred into naïve wt recipients. One day thereafter, mice were infected i.v. with 200 ffu of LCMV‐WE, resulting in an acute infection, and P14 responses were analysed 28 days thereafter (Figure 3B). Overall, P14 cells from H8 and MHCI.GP hosts showed limited expansion, but *Tcf7*‐GFP^+^ cells from MHCI.GP hosts showed a threefold increase in median cell number compared with their *Tcf7*‐GFP^−^ counterpart (Figure 3C), which was stably maintained throughout the course of infection (Figure S4C). When compared with their phenotype at the time of transfer (Figure 3D, ‘pre‐inf’), P14 cells almost completely lost PD1 expression, remained CD44 positive and lost anergy‐associated markers FR4 and CD73, irrespective of their origin and *Tcf7*‐GFP expression at the time of re‐transfer (Figure 3D). Functionally, particularly *Tcf7*‐GFP^+^ P14 cells from MHCI.GP hosts recovered effector capacity to a level comparable to P14 cells from LCMV‐infected wt mice, as indicated by IFN‐γ secretion and degranulation (CD107a expression) in response to *in vitro* restimulation with gp_33‐41_ (Figure 3E). Taken together, these data indicate that TCF1 expression identifies anergic cells that retain an increased ability to expand in response to antigen in the context of a viral infection. Moreover, in line with previous results [7, 42], maintenance of anergy in autoreactive CD8 T cells is dependent on continuous self‐antigen exposure, as in its absence, inflammatory stimuli can reinvigorate effector functions.

### 2.4 Elevated Basal IFN‐I Levels Drive the P14 Cell Phenotype in MHCI.GP Hosts

**FIGURE 4:**
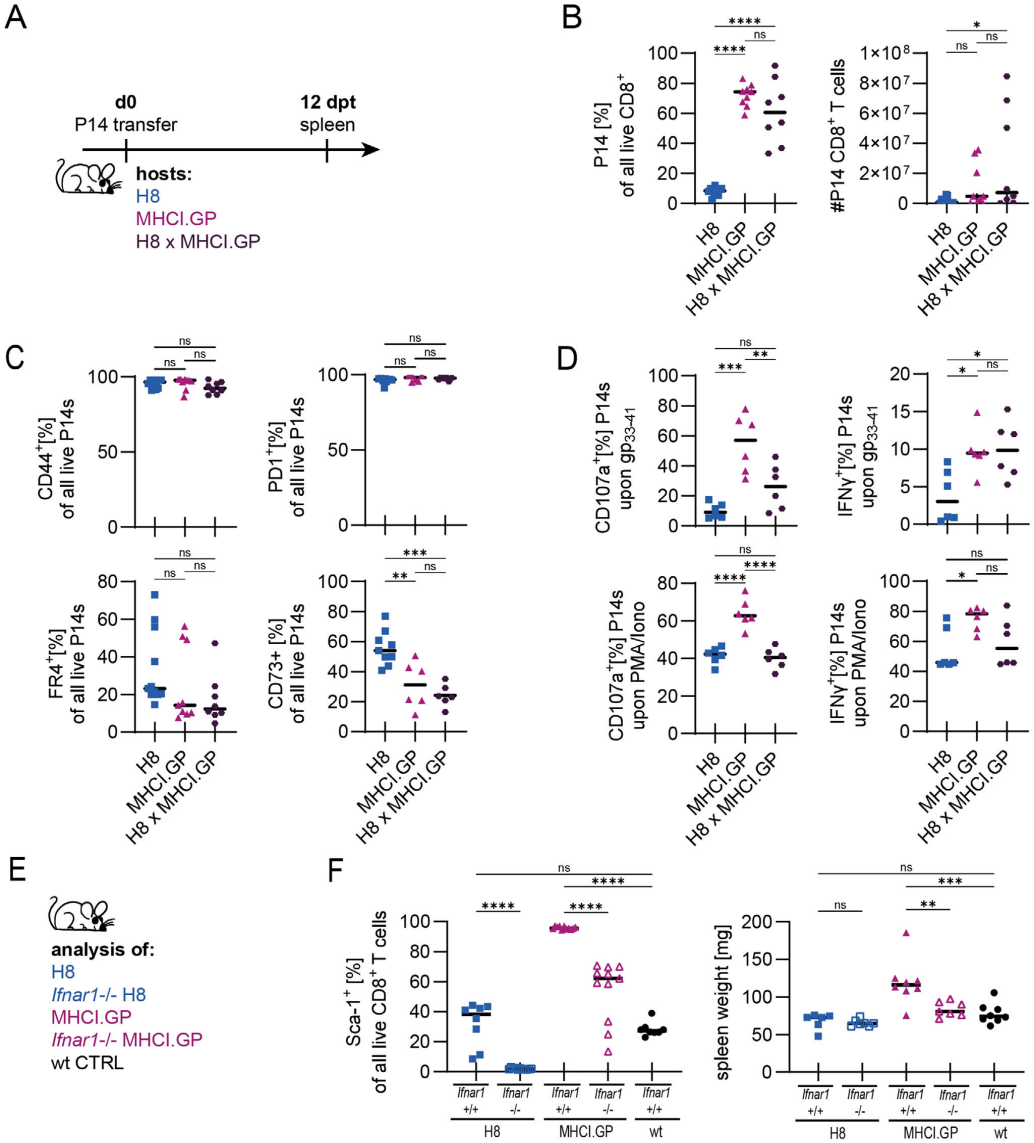
MHCI.GP hosts dominantly shape P14 phenotype via basal IFN‐I. (A) Experimental design. (B) Frequency and number of splenic P14 cells. (C) Proportion of P14 cells from H8 versus MHCI.GP hosts expressing PD1, CD44, FR4 or CD73. (D) IFN‐γ production and CD107a expression of P14 cells isolated from respective hosts and *ex vivo* restimulation with either gp_33‐41_ or PMA/Ionomycin for 6 h. (A–D) Data are pooled from three independent experiments. Data points indicate individual mice, with lines indicating the median data. *p*‐values were calculated with ANOVA with Tukey's post hoc test. ns = not significant, *p < 0.05, **p < 0.01, ***p < 0.001, ****p < 0.0001. (E) Baseline phenotyping of naïve transgenic hosts. F, Sca‐1^+^ expression [%] of all live endogenous CD8 T cells. Data are pooled from two independent experiments. Data points indicate individual mice, with lines indicating the median data. *p*‐values were calculated with ANOVA with Tukey's post hoc test. ns = not significant, **p < 0.01, ***p < 0.001, ****p < 0.0001.

We next set out to understand what drives the fivefold increased expansion potential of P14 cells in MHCI.GP hosts compared with H8 hosts (Figure 1). To test whether differences in antigen expression or the inflammatory environment shape the P14 phenotype, we generated H8 and MHCI.GP double‐transgenic mice and adoptively transferred naïve P14 cells into these hosts (Figure 4A). P14 cells in H8 x MHCI.GP hosts displayed a phenotype resembling cells derived from MHCI.GP mice, including increased expansion (Figure 4B) and reduced upregulation of anergy‐associated markers CD73 and FR4 compared with cells from H8 hosts, while being CD44^+^ and PD1^+^ (Figure 4C). *In vitro* gp_33‐41_ re‐stimulation resulted in elevated IFN‐γ production, while degranulation (CD107a expression) was slightly reduced relative to MHCI.GP hosts (Figure 4D). P14 cells exhibited increased expansion and functionality in mice which presumably expressed the highest levels of self‐antigen (H8 x MHCI.GP) due to co‐expression of the H8 and MHCI.GP transgenes. This observation argues against differences in antigen expression or peptide presentation as the cause for the observed phenotypic differences. We therefore hypothesised that the T cell priming environment present in MHCI.GP transgenic hosts had a dominant effect on the P14 cells’ phenotype.

This hypothesis was supported by a very recent study by Pircher and colleagues showing that transgenic expression of the full‐length LCMV GP in MHCI.GP mice drives elevated basal IFN‐β levels and the upregulation of multiple ISGs [43]. We therefore analysed the expression of stem cell antigen‐1 (Sca‐1; Ly6A/E), a known interferon‐stimulated gene [44, 45, 46], on endogenous CD8 T cells, comparing H8, *Ifnar1* ^−/−^ H8, MHCI.GP, *Ifnar1* ^−/−^ MHCI.GP and wt mice (Figure 4E). Sca‐1 expression was highest in CD8 T cells from MHCI.GP mice, while CD8 T cells from H8 mice expressed Sca‐1 levels comparable to those of wt mice (Figure 4F). IFNAR deletion completely abrogated Sca‐1 expression in H8 mice and reduced Sca‐1 expression in MHCI.GP mice, though it remained increased in comparison to IFNAR sufficient wt and H8 mice. This is likely due to Sca‐1 expression being upregulated upon IFN‐I signalling but also upon sensing of IFN‐γ and IL‐27 [44]. These data are compatible with the finding that LCMV GP transgene expression alters CD4 T cell homeostasis in an IFN‐I‐dependent manner [43]. We concluded that CD8 T cells in MHCI.GP mice are exposed to increased basal inflammation levels, particularly IFN‐I signalling, in comparison to CD8 T cells in H8 mice. This provides a potential explanation for the differential response of adoptively transferred P14 cells into MHCI.GP or H8 hosts.

### 2.5 Direct IFN‐I Sensing by CD8 T Cells Shapes Expansion and Effector Functions Upon Priming by Self‐antigen

**FIGURE 5:**
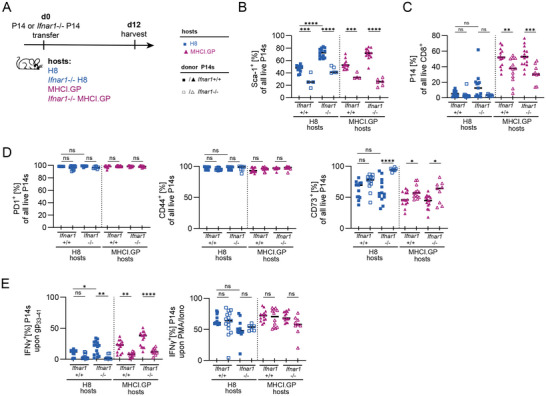
Direct IFN‐I sensing by P14 cells determines expansion and effector function. (A) Experimental design. (B) Sca‐1^+^ expression by adoptively transferred P14 cells. (C) Expansion of P14 cells. (D) Proportion of P14 cells from H8 versus MHCI.GP hosts expressing PD1, CD44, FR4 or CD73. (E) IFN‐γ production of P14 cells isolated from respective hosts and *ex vivo* restimulation with either gp_33‐41_ or PMA/Ionomycin for 6 h. (A–E) Data are pooled from two to three independent experiments. Data points indicate individual mice, with lines indicating the data median. *p*‐values were calculated with ANOVA with Tukey's post hoc test. ns = not significant, *p < 0.05, **p < 0.01, ***p < 0.001, ****p < 0.0001.

A wide range of cells express IFNAR and IFN‐I signalling results in the secretion of immunomodulatory molecules such as cytokines and chemokines, which could indirectly affect CD8 T cells. To dissect whether direct sensing of IFN‐I by P14 cells plays a role, we transferred wt or *Ifnar1* ^−^^/^^−^ P14 cells, identified by CD45.1 or Thy1.1 congenic markers, respectively, into H8, *Ifnar1* ^−^^/^^−^H8, MHCI.GP and *Ifnar1* ^−^^/^^−^MHCI.GP hosts (Figure 5A). Upregulation of Sca‐1 on P14 cells served as a proxy for IFN‐I sensing. P14 cells exhibited the highest expression of Sca‐1 in *Ifnar1* ^−^^/^^−^ H8 and *Ifnar1* ^−^^/^^−^ MHCI.GP hosts (Figure 5B). This is likely the result of increased IFN‐I availability due to the IFNAR deficiency of endogenous cells, making type IFN‐I more readily available for the transferred P14 cells. To investigate this hypothesis and to rule out a potential role for differential TCR signalling as a confounding factor in Sca‐1 expression, we adoptively transferred naïve OT‐I cells, expressing a TCR specific for an ovalbumin‐derived peptide (amino acids 257–264, SIINFEKL) [47] (Figure S5A), into IFNAR sufficient or deficient H8 and MHCI.GP hosts. In H8 recipients, host IFNAR deficiency significantly increased Sca‐1 expression on transferred OT‐I cells, consistent with increased IFN‐I availability. In contrast, OT‐I cells transferred into MHCI.GP hosts exhibited maximal Sca‐1 positivity (>99%) regardless of host genotype (Figure S5B), suggesting that basal IFN‐I levels in IFNAR‐sufficient MHCI.GP are already high enough to drive maximal Sca‐1 induction. These results showed that IFN‐I is directly sensed by CD8 T cells and suggested that variation in IFN‐I signalling may underlie differential anergy of P14 cells in H8 and MHCI.GP mice. In line with this, Sca‐1 expression in P14 cells (Figure 5B) was largely mirrored by P14 expansion (Figure 5C), in that expansion was highest in MHCI.GP hosts and was partially abrogated by IFNAR deficiency in P14 cells. Moreover, while PD1 and CD44 were expressed on almost all P14 cells irrespective of the experimental condition, IFNAR‐deficient P14 cells showed increased expression of anergy‐associated CD73 (Figure 5D) and reduced IFN‐γ production in response to restimulation stimulation with gp_33‐41_ (Figure 5E). IFN‐γ production in response to PMA/Ionomycin stimulation was again comparable between all groups, suggesting that IFN‐I exposure during autoantigen‐mediated priming attenuates the state of anergy, likely by serving as a (partial) signal 3 during the priming process.

Collectively, our results demonstrate that IFN‐I signalling in CD8 T cells during priming to an autoantigen shapes the development of anergy. This has consequences for our understanding of peripheral tolerance as it suggests a model in which increased baseline inflammation, a hallmark of many autoimmune diseases, promotes the generation of anergic self‐reactive cells that express TCF1 and retain an increased capacity to proliferate and exert effector functions, in particular if these cells are re‐activated in the absence of abundant autoantigen exposure. However, as long as the autoantigen is present, effector functions are effectively restrained, highlighting the context‐dependence of anergy‐mediated peripheral tolerance in maintaining immune homeostasis.

## 3 Discussion

Peripheral tolerance is essential to prevent immune responses against autoantigens while allowing the persistence of autoreactive cells, thereby maintaining a broad and protective repertoire. Here, we contribute to the current understanding of peripheral tolerance by revealing that basal IFN‐I levels fine‐tune the fate of naïve CD8 T cells primed by self‐antigens. The strength of TCR signalling is thought to be the primary determinant of autoreactive T cell fate, with stronger stimulation driving T cells towards anergy rather than deletion [6, 7, 11, 48]. Here, we extend this model by adding a role for IFN‐I during priming. Rather than simply overriding the decision between deletion and anergy, IFN‐I acts as a modulatory signal that shapes the quality and persistence of the anergic state. Even upon high‐affinity TCR:antigen interactions, which typically favour deletion or profound anergy, elevated basal IFN‐I signalling preserved a population of anergic CD8 T cells with residual functionality. CD8 T cells primed by their self‐antigen in the presence of low levels of IFN‐I also persisted at higher frequencies and retained more TCF1 expression. This was associated with an increased capacity to exert effector functions in the presence of the autoantigen and with the ability to expand and recover full effector potential upon viral rechallenge in the absence of self‐antigen.

To understand how elevated basal IFN‐I modulates anergy, it is necessary to consider the molecular signalling circuits governing anergy. Classically, anergy is induced when the TCR of naïve CD8 T cells is triggered in the absence of co‐stimulation and inflammation. Such isolated TCR signalling leads to sustained low‐level calcium flux, impaired tyrosine phosphorylation of the TCR ζ‐chain and defective ZAP‐70 activation. This in turn induces profound NFAT activation, while failing to fully induce Ras activation and downstream MAPK signalling, resulting in an imbalance of NFAT, AP‐1 and NF‐κB signalling. In fully activated cells, NFAT and AP‐1 form heterodimers to drive the expression of effector molecules such as IL‐2, while excessive NFAT signalling induces the transcriptional program governing tolerance [49, 50, 51]. Sustained NFAT activity also leads to the upregulation of an inhibitory program, characterised by the induction of PD1 and the transcription factor TOX. Further, NFAT signalling induces the transcription factors EGR2/3 [52], which in turn induce the expression of E3 ubiquitin ligases, such as CBL‐B [53], which actively degrade key proximal TCR signalling components such as PLCγ1 and PKCθ [54, 55]. Additionally, the upregulation of diacylglycerol kinase‐alpha (DGKα) depletes the second messenger DAG, reinforcing the failure of the Ras/MAPK signalling pathway and IL‐2 production [56, 57, 58]. By linking increased IFN‐I signalling to reduced functional CD8 T cell impairment, our data suggest a role for IFN‐I in regulating the induction of this repressive program. By acting as a weak, sustained ‘signal 3’, IFN‐I likely alters the transcriptional landscape before the anergic state is fixed. While not investigated experimentally here, this may involve a shift in STAT utilisation, favouring pro‐survival STAT4/5 signalling over pro‐apoptotic STAT1 pathways [23]. Our data indicate that the magnitude of IFN‐I signalling determines the depth of tolerance, effectively acting as a rheostat. This is demonstrated by comparing autoantigen‐specific CD8 T cells transferred into IFNAR‐deficient H8 mice, where IFN‐I availability is greater than in IFNAR‐sufficient mice, as evidenced by increased Sca‐1 induction. In the presence of such elevated IFN‐I levels, autoantigen‐specific CD8 cells exhibit greater expansion potential and retain higher effector capacity (Figure 5B,C,E). However, it is important to note that IFN‐I levels sufficient to induce ISGs like Sca‐1 and to block cell deletion [59] do not fully override tolerance induction or induce differentiation into effector cells. IFN‐I likely achieves this by reducing the induction of anergy‐enforcing factors (such as TOX, EGR2/3 or CBL‐B) to limit a proximal signalling block without breaking tolerance. Thus, IFN‐I does not act as a binary switch but rather fine‐tunes the extent of tolerance induction in a dose‐dependent manner.

Modulation of anergy by low levels of IFN‐I stands in contrast to autoreactive cells primed in the presence of high levels of inflammation. For example, LPS exposure and IL‐12 have been shown to interfere with tolerance induction [12, 13, 29]. In line with a previous study by Curtsinger et al. [12], we find that priming by self‐antigens in the presence of inflammation allows CD8 T cells to expand. Here, we propose that low‐level IFN‐I signalling actively shapes the anergic state. This is underscored by changes in proximal TCR signalling and anergy‐associated markers such as PD1, CD73 and FR4. We therefore propose that early exposure to IFN‐I alters the differentiation trajectory of tolerised CD8 T cells.

Peripheral tolerance is increasingly recognised as a distinct differentiation trajectory that diverges early from effector fates and that is enforced by changes in protein translation [32]. Typically, tolerant CD8 T cells fail to upregulate the ribosomal machinery necessary for clonal expansion [60]. However, increased expression of the stem‐like transcription factor TCF1 may preserve translational capacity. TCF1^+^ stem‐like CD8 T cells have been shown to maintain expression of Wnt‐target genes, including the master metabolic regulator MYC [61]. MYC is a key driver of glutaminolysis and metabolic reprogramming [62], and increased MYC expression in tolerised CD8 T cells has been associated with increased protein translation capacity and breach of tolerance [32]. Inflammatory stimuli also act post‐transcriptionally to stabilize MYC [63] and can promote epigenetic changes that sustain protein translation. While previous literature does not suggest that IFN‐I directly promotes TCF‐1 expression [24, 64], IFN‐I may influence these mechanisms to preserve CD8 T cell function, given its ability to regulate mRNA translation via PI3K and mTOR signalling [21, 22].

Importantly, the full effector potential, specifically robust IFN‐γ production in CD8 T cells differentiated from TCF1^+^ CD8 T cells primed in the presence of elevated basal IFN‐I levels, was only regained following re‐transfer into recipient mice that did not express the self‐antigen. This setting mimics, to some extent, a physiological setting in which CD8 T cells recirculate between tissues that do or do not express a particular autoantigen. We propose that such migration can provide a temporary ‘antigen holiday’ for autoreactive CD8 T cells. This is supported by recent findings in T1D, which identified a stem‐like autoimmune progenitor population that resides in the antigen‐restricted niche of the pancreatic‐draining lymph node rather than in the antigen‐rich pancreas. This ‘holiday’ from chronic TCR stimulation in the tissue was shown to be essential for replenishing short‐lived effector cells that mediate autoimmune pathology [61].

By identifying basal IFN‐I as a critical gatekeeper that diverts autoreactive clones away from dysfunction, the results presented here have significant implications for our understanding of autoimmune diseases. This mechanism is particularly relevant to pathologies characterised by elevated type I interferon signatures, such as SLE, where a failure to delete autoreactive clones contributes to disease persistence [26, 65]. Furthermore, considering the rising prevalence of systemic inflammation driven by dietary habits and metabolic dysregulation, our data are in line with a model in which such chronic, low‐grade cytokine release favours the accumulation of autoreactive CD8 T cells with less robust anergy and hence increased effector functions. Unlike terminally exhausted or deleted cells, these T cells remain functionally restrained in the presence of autoantigen, yet poised to break tolerance upon subsequent triggers in the absence of autoantigen. Consequently, our data provide a mechanistic rationale for therapeutic strategies targeting the IFN‐I signalling axis in autoimmunity.

## 4 Data Limitations and Perspectives

While we establish IFN‐I as a critical rheostat fine‐tuning the fate of autoreactive CD8 T cells, the precise downstream signalling events linking IFN‐I to the state of anergy remain unresolved. For example, future studies should investigate whether IFN‐I influences the balance of STAT4/5 and STAT1 pathways. Furthermore, our model proposes that the functional plasticity of CD8 T cells relies on maintaining TCF1. While supported by previous research showing a strong association between TCF1 and metabolic fitness, we did not directly assess metabolic flux or protein translation rates. Finally, our study relies on the use of experimental animal models to allow insights into peripheral CD8 T cell tolerance, and we cannot fully exclude that there are other factors, besides the levels of IFN‐I, that differ between H8 and MHCI.GP hosts.

## 5 Materials and Methods

### 5.1 Mice

Mice were bred and maintained under specific‐pathogen‐free conditions at the ETH Phenomics Center Hönggerberg. They were housed in standard cages in groups of up to five animals, with a 12 h light–dark cycle, an ambient temperature of 22 ± 2°C, and humidity maintained at 55 ± 10%. Male and female mice aged 6 to 20 weeks were used for the experiments in this study. Transgenic MHCI.GP [30], H8 [29], *Ifnar1* ^−^^/^^−^ [66], *Nr4a1‐*GFP mice [33] expressing GFP under the control of the *Nr4a1* promoter, *Tcf7‐*GFP mice [41] expressing GFP under the control of *Tcf7* promoter, P14 (CD45.1) mice expressing a TCR specific for LCMV peptide gp_33‐41_ (KAVYNFATC) [67] and OT‐I (CD45.1) mice expressing a TCR specific for the ovalbumin‐derived peptide OVA_257‐264_ (SIINFEKL) [47] were described previously. As controls, wt littermates, or wt mice obtained from the ETH Phenomics Center and Janvier Labs were used.

### 5.2 P14 isolation and Adoptive Transfers

For adoptive transfers, naïve CD45.1^+^ P14 cells or naïve Thy1.1^+^ P14 cells were isolated from spleens and lymph nodes using the EasySep Mouse Naïve CD8^+^ T Cell Isolation Kit (STEMCELL) according to the manufacturer's instructions. Unless stated otherwise, cells were resuspended in PBS (500,000 cells/200 µL) and intravenously injected into respective CD45.2^+^ recipient mice.

### 5.3 Viruses and Infections

Acute LCMV infections were conducted by injecting mice intravenously with 200 ffu LCMV‐WE. For MCMV‐ie2‐gp33 infections, mice were injected i.v. with 2 × 10^5^ pfu. Stocks were propagated as previously described [68].

### 5.4 Flow Cytometry

Staining for flow cytometry analysis of blood samples and splenocytes was performed according to guidelines for the use of flow cytometry in immunological studies [69]. Spleens were harvested and mashed through 70 µm strainers (Miltenyi Biotec) to create single‐cell suspensions. Erythrocyte lysis of single‐cell suspensions (ACK lysis treatment for 5 min at RT) was performed before surface staining for 20 min at 4°C, with concentrations pre‐determined through antibody titrations. Fluorescently labelled antibodies specific for CD107a (clone 1D4B; APC or PE; BioLegend), CD44 (clone IM7; APC, BV510, BV785, FITC, or PE; BioLegend), CD45.1 (clone A20; AF700, APC, BV711, BV785, Pacific Blue, PE, or PE‐Dazzle; BioLegend), CD45.2 (clone 104; BUV737 and BUV805; BD Biosciences; Pacific Blue and PE‐Cy7; BioLegend), CD73 (clone Ty/11.8; BV605; BioLegend), CD8 (clone 53–6.7; BUV395; BD Biosciences and BV605, BV650, BV711, PerCP, or Spark Blue 574; BioLegend), FR4 (clone 12A5; PerCP‐Cy5.5 or RY586; BioLegend), PD‐1 (clone 29F.1A12; BV605, PE‐Cy7, or PE‐Fire 810; BioLegend), Sca‐1/Ly6A/E (clone D7; AF488 or AF647; BioLegend) and Thy1.1 (clone OX‐7; PerCP; BioLegend; or clone HIS51; PE; eBiosciene) were used. Cell viability was determined using a LIVE/DEAD Fixable Dead Cell Stain Kit (Aqua, Blue, NIR or Scarlet; Invitrogen). For intracellular staining, cells were further processed using the Foxp3/Transcription Factor Staining Buffer Set (eBioscience) according to the manufacturer's instructions, followed by intracellular staining for 1 h at RT. For intracellular cytokine staining, IFN‐γ (clone XMG1.2; BV421 or PE‐Cy7; BioLegend) and TNF‐α (clone MP6‐XT22; BV650; BioLegend) antibodies were used. For Ca^2+^ Flux analysis, cells were labelled with Fura Red (Invitrogen) according to the manufacturer's instructions and left either unstimulated or stimulated during acquisition. Data were acquired on a BD LSRFortessa or BD FACSymphony A5 SE cell analyser, and fluorescence‐activated cell sorting was performed on a BD FACSAria cell sorter, running on the BD FACSDiva Software. Data was analysed using the FlowJo software (Version 10.10). Samples were gated according to Figure S6.

### 5.5 *In Vitro* Assays

To investigate cytokine production *in vitro*, splenocytes were cultured in complete RPMI (RPMI‐1640, BioConcept) containing 2 mM L‐glutamine (BioConcept), 2% penicillin‐streptomycin (Sigma‐Aldrich), 1x Non‐Essential Amino Acids (Sigma‐Aldrich), 1 mM sodium pyruvate (Gibco), 10% fetal bovine serum (Omnilab), 25 mM HEPES (Gibco), 50 µM β‐Mercaptoethanol (Gibco) in the presence of a protein transport inhibitor (eBioscience) and anti‐mouse CD107a (BioLegend) at 37°C for 6 h. Cells were either stimulated with 1 µg/mL gp_33‐41_ (KAVYNFATC) peptide (EMC Microcollections) or PMA/Ionomycin (eBioscience Cell Stimulation Cocktail) according to the manufacturer's instructions.

### 5.6 Statistical Analysis

Statistical Analysis was performed using GraphPad Prism Software, Version 10.4. Statistical tests and significance levels are indicated in each figure.

## Author Contributions

Anne S. Haefke, Roman Spörri and Annette Oxenius designed the experiments. Anne S. Haefke, Hanna Gröber, Ioana Sandu, Vanessa Skipness, Nikos Pantouloufos, Fabienne Gräbnitz, Marie‐Elen Tuchel, Nathan Zangger, Dominique Stark, Marvin Kříž and Carmine Cristinzio performed the experiments. Anne S. Haefke, Roman Spörri and Annette Oxenius analysed the experiments. Hanspeter Pircher provided reagents and edited the manuscript. Anne S. Haefke, Isaak Quast and Annette Oxenius wrote the manuscript.

## Ethics Approval for Animal Studies

All animal experiments were conducted according to the Swiss federal regulations and were approved by the Cantonal Veterinary Office of Zürich (Animal experimentation permissions ZH022/20 and ZH097/23).

## Conflicts of Interest

The authors declare no conflicts of interest.

## Supporting information




**Supporting File 1**: eji70192‐sup‐0001‐Figure‐Legends.docx.

**Supporting File 2**: eji70192‐sup‐0002‐Figures.pdf.

## Data Availability

Further information on data, resources and reagents will be provided by the corresponding author upon request.

## References

[1] A. M. Gallegos and M. J. Bevan , “Central Tolerance: Good but Imperfect,” Immunological Reviews 209 (2006): 290–296, 10.1111/j.0105-2896.2006.00348.x. 16448550

[2] W. Yu , N. Jiang , P. J. R. Ebert , et al., “Clonal Deletion Prunes but Does Not Eliminate Self‐Specific Αβ CD8+ T Lymphocytes,” Immunity 42 (2015): 929–941, 10.1016/J.IMMUNI.2015.05.001. 25992863 PMC4455602

[3] K. Wing and S. Sakaguchi , “Regulatory T Cells Exert Checks and Balances on Self Tolerance and Autoimmunity,” Nature Immunology 11 (2009): 7–13, 10.1038/ni.1818. 20016504

[4] J. Hernandez , S. Aung , W. L. Redmond , and L. A. Sherman , “Phenotypic and Functional Analysis of CD8(+) T Cells Undergoing Peripheral Deletion in Response to Cross‐presentation of Self‐antigen,” Journal of Experimental Medicine 194 (2001): 707–717, 10.1084/JEM.194.6.707. 11560988 PMC2195957

[5] C. Kurts , H. Kosaka , F. R. Carbone , J. Miller , and W. R. Heath , “Class I–restricted Cross‐Presentation of Exogenous Self‐Antigens Leads to Deletion of Autoreactive CD8+ T Cells,” Journal of Experimental Medicine 186 (1997): 239–245, 10.1084/JEM.186.2.239. 9221753 PMC2198972

[6] W. L. Redmond , B. C. Marincek , and L. A. Sherman , “Distinct Requirements for Deletion versus Anergy During CD8 T Cell Peripheral Tolerance in Vivo,” The Journal of Immunology 174 (2005): 2046–2053, 10.4049/jimmunol.174.4.2046. 15699134

[7] W. L. Redmond and L. A. Sherman , “Peripheral Tolerance of CD8 T Lymphocytes,” Immunity 22 (2005): 275–284, 10.1016/j.immuni.2005.01.010. 15780985

[8] A. Schietinger , J. J. Delrow , R. S. Basom , J. N. Blattman , and P. D. Greenberg , “Rescued Tolerant CD8 T Cells Are Preprogrammed to Reestablish the Tolerant state,” Science 335 (2012): 723–727, 10.1126/SCIENCE.1214277/SUPPL_FILE/SCHIETINGER.SOM.PDF. 22267581 PMC3754789

[9] R. H. Schwartz , “T Cell Anergy *,” Annual Review of Immunology 21 (2003): 305–339, 10.1146/annurev.immunol.21.120601.141110. 12471050

[10] M. A. ElTanbouly and R. J. Noelle , “Rethinking Peripheral T Cell Tolerance: Checkpoints Across a T Cell's Journey,” Nature Reviews Immunology 21 (2020): 257–267, Available at: https://www.nature.com/articles/s41577‐020‐00454‐2 [Accessed June 2, 2022]. 10.1038/s41577-020-00454-2 PMC12536352 33077935

[11] B. Rocha , A. Grandien , and A. A. Freitas , “Anergy and Exhaustion Are Independent Mechanisms of Peripheral T Cell Tolerance,” Journal of Experimental Medicine 181 (1995): 993–1003, 10.1084/jem.181.3.993. 7869056 PMC2191934

[12] J. M. Curtsinger , D. C. Lins , and M. F. Mescher , “Signal 3 Determines Tolerance versus Full Activation of Naive CD8 T Cells Dissociating Proliferation and Development of Effector Function,” Journal of Experimental Medicine 197 (2003): 1141–1151, 10.1084/JEM.20021910. 12732656 PMC2193970

[13] J. M. Curtsinger , J. O. Valenzuela , P. Agarwal , D. Lins , and M. F. Mescher , “Cutting Edge: Type I IFNs Provide a Third Signal to CD8 T Cells to Stimulate Clonal Expansion and Differentiation,” The Journal of Immunology 174 (2005): 4465–4469, 10.4049/JIMMUNOL.174.8.4465. 15814665

[14] M. Srinivasan and K. A. Frauwirth , “Peripheral Tolerance in CD8+ T Cells,” Cytokine 46 (2009): 147–159, 10.1016/j.cyto.2009.01.010. 19268604

[15] J. Hernández , S. Aung , K. Marquardt , and L. A. Sherman , “Uncoupling of Proliferative Potential and Gain of Effector Function by CD8(+) T Cells Responding to Self‐antigens,” Journal of Experimental Medicine 196 (2002): 323–333, 10.1084/JEM.20011612. 12163561 PMC2193940

[16] G. A. Kolumam , S. Thomas , L. J. Thompson , J. Sprent , and K. Murali‐Krishna , “Type I Interferons Act Directly on CD8 T Cells to Allow Clonal Expansion and Memory Formation in Response to Viral Infection,” Journal of Experimental Medicine 202 (2005): 637–650, 10.1084/JEM.20050821. 16129706 PMC2212878

[17] P. Aichele , H. Unsoeld , M. Koschella , O. Schweier , U. Kalinke , and S. Vucikuja , “CD8 T Cells Specific for Lymphocytic Choriomeningitis Virus Require Type I IFN Receptor for Clonal Expansion,” Journal of Immunology 176 (2006): 4525–4529, 10.4049/JIMMUNOL.176.8.4525. 16585541

[18] M. Wiesel , J. Crouse , G. Bedenikovic , A. Sutherland , N. Joller , and A. Oxenius , “Type‐I IFN Drives the Differentiation of Short‐Lived Effector CD8+ T Cells in Vivo,” European Journal of Immunology 42 (2012): 320–329, 10.1002/EJI.201142091. 22102057

[19] G. R. Stark , I. M. Kerr , B. R. G. Williams , R. H. Silverman , and R. D. Schreiber , “How Cells Respond to Interferons,” Annual Review of Biochemistry 67 (1998): 227–264, 10.1146/ANNUREV.BIOCHEM.67.1.227. 9759489

[20] J. W. Schoggins , “Interferon‐Stimulated Genes: What Do They All Do?,” Annual Review of Virology 6 (2019): 567–584, 10.1146/ANNUREV-VIROLOGY-092818-015756. 31283436

[21] R. H. Lurie and L. C. Platanias , “Mechanisms of Type‐I‐ and Type‐II‐Interferon‐Mediated Signalling,” Nature Reviews Immunology 5 (2005): 375–386, 10.1038/nri1604. 15864272

[22] C. Mazewski , R. E. Perez , E. N. Fish , and L. C. Platanias , “Type I Interferon (IFN)‐Regulated Activation of Canonical and Non‐Canonical Signaling Pathways,” Frontiers in Immunology 11 (2020): 606456, 10.3389/FIMMU.2020.606456/FULL. 33329603 PMC7719805

[23] J. Crouse , U. Kalinke , and A. Oxenius , “Regulation of Antiviral T Cell Responses by Type I Interferons,” Nature Reviews Immunology 15 (2015): 231–242, 10.1038/nri3806. 25790790

[24] T. Wu , Y. Ji , E. Ashley Moseman , et al., “The TCF1‐Bcl6 Axis Counteracts Type I Interferon to Repress Exhaustion and Maintain T Cell Stemness,” Science Immunology 1 (2016), 10.1126/SCIIMMUNOL.AAI8593. PMC5179228 28018990

[25] D. J. Gough , N. L. Messina , C. J. P. Clarke , R. W. Johnstone , and D. E. Levy , “Constitutive Type I Interferon Modulates Homeostatic Balance Through Tonic Signaling,” Immunity 36 (2012): 166–174, 10.1016/j.immuni.2012.01.011. 22365663 PMC3294371

[26] J. C. Hall and A. Rosen , “Type I Interferons: Crucial Participants in Disease Amplification in Autoimmunity,” National Review of Rheumatology 6 (2010): 40–49, 10.1038/NRRHEUM.2009.237. PMC3622245 20046205

[27] L. B. Ivashkiv and L. T. Donlin , “Regulation of Type i Interferon Responses,” Nature Reviews Immunology 14 (2014): 36–49, 10.1038/NRI3581;SUBJMETA. PMC4084561 24362405

[28] H. Pircher , K. Bürki , R. Lang , H. Hengartner , and R. M. Zinkernagel , “Tolerance Induction in Double Specific T‐Cell Receptor Transgenic Mice Varies With Antigen,” Nature 342 (1989): 559–561, 10.1038/342559a0. 2573841

[29] S. Ehl , J. Hombach , P. Aichele , et al., “Viral and Bacterial Infections Interfere With Peripheral Tolerance Induction and Activate CD8+ T Cells to Cause Immunopathology,” Journal of Experimental Medicine 187 (1998): 763–774, 10.1084/jem.187.5.763. 9480986 PMC2212172

[30] C. Woopen , T. Straub , O. Schweier , et al., “Immunological Tolerance to LCMV Antigens Differently Affects Control of Acute and Chronic Virus Infection in Mice,” European Journal of Immunology 48 (2018): 120–127, 10.1002/eji.201747156. 28921501

[31] R. M. Teague , P. D. Greenberg , C. Fowler , et al., “Peripheral CD8+ T Cell Tolerance to Self‐Proteins Is Regulated Proximally at the T Cell Receptor,” Immunity 28 (2008): 662–674, 10.1016/J.IMMUNI.2008.03.012. 18424189 PMC3443683

[32] B. W. V. Der , N. S , T. J. Peters , et al., “The CD8+ T Cell Tolerance Checkpoint Triggers a Distinct Differentiation state Defined by Protein Translation Defects,” Immunity 0 (2024), 10.1016/J.IMMUNI.2024.04.026. PMC11807353 38776918

[33] A. E. Moran , K. L. Holzapfel , Y. Xing , et al., “T Cell Receptor Signal Strength in Treg and iNKT Cell Development Demonstrated by a Novel Fluorescent Reporter Mouse,” Journal of Experimental Medicine 208 (2011): 1279–1289, 10.1084/JEM.20110308. 21606508 PMC3173240

[34] F. Tsushima , S. Yao , T. Shin , et al., “Interaction Between B7‐H1 and PD‐1 Determines Initiation and Reversal of T‐cell Anergy,” Blood 110 (2007): 180–185, 10.1182/BLOOD-2006-11-060087. 17289811 PMC1896111

[35] I. A. Parish , S. Rao , G. K. Smyth , et al., “The Molecular Signature of CD8 T Cells Undergoing Deletional Tolerance,” (2009), 10.1182/blood-2008-10. PMC2680364 19204323

[36] J. Hernandez , S. Aung , W. L. Redmond , and L. A. Sherman , “Phenotypic and Functional Analysis of CD8(+) T Cells Undergoing Peripheral Deletion in Response to Cross‐presentation of Self‐antigen,” Journal of Experimental Medicine 194 (2001): 707–717, 10.1084/JEM.194.6.707. 11560988 PMC2195957

[37] H. C. Probst , K. McCoy , T. Okazaki , T. Honjo , and M. Van Den Broek , “Resting Dendritic Cells Induce Peripheral CD8+ T Cell Tolerance Through PD‐1 and CTLA‐4,” Nature Immunology 6 (2005): 280–286, 10.1038/NI1165. 15685176

[38] I. A. Parish , S. Rao , G. K. Smyth , et al., “The Molecular Signature of CD8 T Cells Undergoing Deletional Tolerance,” Blood (2009), 10.1182/blood-2008-10. PMC2680364 19204323

[39] S. P. M. Welten , A. Redeker , K. Franken , et al., “The Viral Context Instructs the Redundancy of Costimulatory Pathways in Driving CD8+ T Cell Expansion,” Elife 4 (2015): e07486, 10.7554/ELIFE.07486. 26263500 PMC4558566

[40] X. Zhou , S. Yu , D. M. Zhao , J. T. Harty , V. P. Badovinac , and H. H. Xue , “Differentiation and Persistence of Memory CD8+ T Cells Depend on T Cell Factor 1,” Immunity 33 (2010): 229–240, 10.1016/j.immuni.2010.08.002. 20727791 PMC2928475

[41] D. T. Utzschneider , M. Charmoy , V. Chennupati , et al., “T Cell Factor 1‐Expressing Memory‐Like CD8+ T Cells Sustain the Immune Response to Chronic Viral Infections,” Immunity 45 (2016): 415–427, 10.1016/J.IMMUNI.2016.07.021. 27533016

[42] B. Rocha , C. Tanchot , and B. H. Von , “Clonal Anergy Blocks in Vivo Growth of Mature T Cells and Can Be Reversed in the Absence of Antigen,” Journal of Experimental Medicine 177 (1993): 1517–1521, 10.1084/jem.177.5.1517. 8478622 PMC2191004

[43] H. Pircher , O. S. Thomas , A. S. Haefke , A. Oxenius , and T. Boehm , “Increased Constitutive Interferon‐β Levels and Altered CD4 T Cell Homeostasis Induced by Expression of a Viral Glycoprotein,” European Journal of Immunology 56 (2026): e70188, 10.1002/EJI.70188. 41972454 PMC13073064

[44] J. H. Delong , A. O. Hall , C. Konradt , et al., “Cytokine‐ and TCR‐Mediated Regulation of T Cell Expression of Ly6C and Sca‐1,” The Journal of Immunology 200 (2018): 1761–1770, 10.4049/JIMMUNOL.1701154. 29358280 PMC5821564

[45] F. J. Dumont , R. Dijkmans , R. G. E. Palfree , R. D. Boltz , and L. Coker , “Selective Up‐Regulation by Interferon‐γ of Surface Molecules of the Ly‐6 Complex in Resting T Cells: The Ly‐6A/E and TAP Antigens Are Preferentially Enhanced,” European Journal of Immunology 17 (1987): 1183–1191, 10.1002/eji.1830170816. 3040423

[46] C. Holmes and W. L. Stanford , “Concise Review: Stem Cell Antigen‐1: Expression, Function, and Enigma,” Stem Cells 25 (2007): 1339–1347, 10.1634/STEMCELLS.2006-0644. 17379763

[47] K. A. Hogquist , S. C. Jameson , W. R. Heath , J. L. Howard , M. J. Bevan , and F. R. Carbone , “T Cell Receptor Antagonist Peptides Induce Positive Selection,” Cell 76 (1994): 17–27, 10.1016/0092-8674(94)90169-4. 8287475

[48] G. M. Davey , C. Kurts , J. Miller , et al., “Peripheral Deletion of Autoreactive CD8 T Cells by Cross Presentation of Self‐antigen Occurs by a Bcl‐2‐Inhibitable Pathway Mediated by Bim,” Journal of Experimental Medicine 196 (2002): 947–955, 10.1084/jem.20020827. 12370256 PMC2194028

[49] N. Soto‐Nieves , I. Puga , B. T. Abe , et al., “Transcriptional Complexes Formed by NFAT Dimers Regulate the Induction of T Cell Tolerance,” Journal of Experimental Medicine 206 (2009): 867–876, 10.1084/JEM.20082731. 19307325 PMC2715123

[50] F. Macián , F. García‐Cózar , S. H. Im , H. F. Horton , M. C. Byrne , and A. Rao , “Transcriptional Mechanisms Underlying Lymphocyte Tolerance,” Cell 109 (2002): 719–731, 10.1016/S0092-8674(02)00767-5. 12086671

[51] P. G. Hogan , L. Chen , J. Nardone , and A. Rao , “Transcriptional Regulation by Calcium, Calcineurin, and NFAT,” Genes & Development 17 (2003): 2205–2232, 10.1101/GAD.1102703. 12975316

[52] M. Safford , S. Collins , M. A. Lutz , et al., “Egr‐2 and Egr‐3 Are Negative Regulators of T Cell Activation,” Nature Immunology 6 (2005): 472–480, 10.1038/NI1193. 15834410

[53] Y. J. Chiang , H. K. Kole , K. Brown , et al., “Cbl‐b Regulates the CD28 Dependence of T‐Cell Activation,” Nature 403 (2000): 216–220, 10.1038/35003235. 10646609

[54] F. Macián , S. H. Im , F. J. García‐Cózar , and A. Rao , “T‐Cell Anergy,” Current Opinion in Immunology 16 (2004): 209–216, 10.1016/J.COI.2004.01.013. 15023415

[55] M. S. Jeon , A. Atfield , K. Venuprasad , et al., “Essential Role of the E3 Ubiquitin Ligase Cbl‐b in T Cell Anergy Induction,” Immunity 21 (2004): 167–177, 10.1016/j.immuni.2004.07.013. 15308098

[56] C. G. Fathman and N. B. Lineberry , “Molecular Mechanisms of CD4+ T‐Cell Anergy,” Nature Reviews Immunology 7 (2007): 599–609, 10.1038/NRI2131. 17612584

[57] B. A. Olenchock , R. Guo , J. H. Carpenter , et al., “Disruption of Diacylglycerol Metabolism Impairs the Induction of T Cell Anergy,” Nature Immunology 7 (2006): 1174–1181, 10.1038/NI1400. 17028587

[58] P. Chappert and R. H. Schwartz , “Induction of T Cell Anergy: Integration of Environmental Cues and Infectious Tolerance,” Current Opinion in Immunology 22 (2010): 552–559, 10.1016/J.COI.2010.08.005. 20869863 PMC2981408

[59] G. Lombardi , P. J. Dunne , D. Scheel‐Toellner , et al., “Type 1 IFN Maintains the Survival of Anergic CD4+ T Cells,” The Journal of Immunology 165 (2000): 3782–3789, 10.4049/JIMMUNOL.165.7.3782. 11034383

[60] T. C. J. Tan , J. Knight , T. Sbarrato , K. Dudek , A. E. Willis , and R. Zamoyska , “Suboptimal T‐Cell Receptor Signaling Compromises Protein Translation, Ribosome Biogenesis, and Proliferation of Mouse CD8 T Cells,” Proceedings of National Academy of Science 114 (2017): E6117–E6126, 10.1073/PNAS.1700939114/SUPPL_FILE/PNAS.201700939SI.PDF. PMC5544288 28696283

[61] S. V. Gearty , F. Dündar , P. Zumbo , et al., “An Autoimmune Stem‐Like CD8 T Cell Population Drives Type 1 Diabetes,” Nature 602 (2021): 156–161, 10.1038/s41586-021-04248-x. 34847567 PMC9315050

[62] H. Wang , X. Feng , W. Yan , and D. Tian , “Regulatory T Cells in Autoimmune Hepatitis: Unveiling Their Roles in Mouse Models and Patients,” Frontiers in Immunology 11 (2020): 575572, 10.3389/FIMMU.2020.575572/XML. 33117375 PMC7575771

[63] G. C. Preston , L. V. Sinclair , A. Kaskar , et al., “Single Cell Tuning of Myc Expression by Antigen Receptor Signal Strength and Interleukin‐2 in T Lymphocytes,” Embo Journal 34 (2015): 2008–2024, 10.15252/embj.201490252/SUPPL_FILE/EMBJ201490252.REVIEWER_COMMENTS.PDF. 26136212 PMC4551349

[64] M. Danilo , V. Chennupati , J. G. Silva , S. Siegert , and W. Held , “Suppression of Tcf1 by Inflammatory Cytokines Facilitates Effector CD8 T Cell Differentiation,” Cell reports 22 (2018): 2107–2117, 10.1016/j.celrep.2018.01.072. 29466737

[65] M. Shrivastav and T. B. Niewold , “Nucleic Acid Sensors and Type I Interferon Production in Systemic Lupus Erythematosus,” Frontiers in immunology 4 (2013): 319, 10.3389/FIMMU.2013.00319. 24109483 PMC3791549

[66] U. Müller , U. Steinhoff , L. F. L. Reis , et al., “Functional Role of Type I and Type II Interferons in Antiviral Defense,” Science 264 (1994): 1918–1921, 10.1126/SCIENCE.8009221. 8009221

[67] H. Pircher , D. Moskophidis , U. Rohrer , K. Bürki , H. Hengartner , and R. M. Zinkernagel , “Viral Escape by Selection of Cytotoxic T Cell‐Resistant Virus Variants in Vivo,” Nature 346 (1990): 629–633, 10.1038/346629a0. 1696684

[68] S. P. M. Welten , J. Oderbolz , V. Yilmaz , et al., “Influenza‐ and MCMV‐induced Memory CD8 T Cells Control respiratory Vaccinia Virus Infection Despite Residence in Distinct Anatomical Niches,” Mucosal Immunology 14 (2021): 728–742, 10.1038/s41385-020-00373-4. 33479479 PMC8075924

[69] A. Cossarizza , H. D. Chang , A. Radbruch , et al., “Guidelines for the Use of Flow Cytometry and Cell Sorting in Immunological Studies (Third Edition),” European Journal of Immunology 51 (2021): 2708–3145, 10.1002/EJI.202170126. 34910301 PMC11115438

