# Supplementary material for: CD8 T Cell Sensing of Type I Interferon Impacts Anergy
Source: Eur J Immunol. 2026 May 7;56:e70192. doi: 10.1002/eji.70192 (PMC13150955; doi:10.1002/eji.70192)
Supplement: Supplementary file 1 — Supporting File 1: eji70192‐sup‐0001‐Figure‐Legends.docx. [file EJI-56-e70192-s002.docx]

## Figure Legends Supplementary

**Supp. Fig. 1|Adoptive P14 cell transfer into H8 and MHCI.GP hosts does not result in clinical pathology. A,** Experimental design. **B,** Serum ALT [U/l] measured 12 days post adoptive transfer. **C,** Body weight measurement throughout experiment. Data are shown as individual biological replicates + mean. If applicable, p values were calculated with ANOVA with Tukey’s post hoc test. ns=not significant.

**Supp. Fig. 2|** **Anergic phenotype of P14 cells induced in H8 and MHCI.GP is stably retained for at least 30 days. A,** Experimental design. Analysis of splenocytes 30 days post adoptive transfer of naïve P14 cells**. B,** Frequency and number of splenic P14 cells. **C,** Representative flow cytometry plots showing the frequency of PD1, CD44, FR4 and CD73 expression among P14 cells and **D,** Quantification. **E**, Representative flow cytometry plots showing IFNγ production and degranulation (CD107a) by P14 cells from H8 or MHCI.GP hosts following 6h *ex vivo* restimulation with either gp_33-41_ or PMA/Ionomycin and **F,** Quantification. **A-F**, Data show three biological replicates per group from one experimental repeat. **B,+D,+F,** Data are shown as individual biological replicates + mean. P values were calculated with an unpaired t-test. *p<0.05, **p<0.01.

**Supp. Fig. 3| Proximal TCR signalling in P14 cells from H8 and MHCI.GP hosts. A,** Experimental design. Analysis of splenocytes 12 days post adoptive transfer of naïve P14 cells**. B,** Ca^2+^ flux by P14s isolated from respective hosts at baseline (0-60s) and in response to stimulation (60s-160s) with either anti-CD3/CD28 (top panels) or PMA/Ionomycin (bottom panels). Lines show two biological replicates. **C,** Experimental design. Analysis of splenocytes was performed 12 days post adoptive transfer of naïve *Nr4a1*-GFP reporter P14 cells (H8, MHCI.GP hosts, left schematic) or 10 days after acute LCMV infection (wt hosts, right schematic)**. D,** *Nr4a1*-GFP (geometric MFI) expression without additional stimulation. **E,** Representative *Nr4a1*-GFP expression upon TCR stimulation via DCs loaded with gp_33-41_, PMA-Ionomycin, aCD3CD28, or without additional stimulation. Black lines depict P14 cells from infected wt hosts; magenta: MHCI.GP; blue: H8. Data show two biological replicates per group from one experimental repeat. Data are shown as individual biological replicates + mean.

**Supp. Fig. 4|** ***Tcf7*-GFP⁺ or *Tcf7*-GFP⁻ P14 cell subsets from H8 versus MHCI.GP hosts are phenotypically similar but** ***Tcf7*-GFP^+^ P14 cells have superior expansion potential upon retransfer into antigen-free environment and infection.** **A**, Proportion of naïve, *Tcf7*-GFP⁺ or *Tcf7*-GFP⁻ P14 cell subsets from H8 versus MHCI.GP hosts expressing CD73, FR4 or PD1. Data are pooled from two independent experiments. Data points indicate individual mice, with lines indicating data median. P values were calculated with ANOVA with Tukey’s post hoc test. ns=not significant, ***p<0.001. **B,** Gating strategy for sorting of *Tcf7*-GFP⁺ or *Tcf7*-GFP⁻ P14 cell subsets from H8 and MHCI.GP hosts. **C**, Expansion of different P14 cell subsets after retransfer into wt hosts and acute LCMV-WE infection. Data are pooled from two independent experiments, lines indicating data mean + SD.

**Supp. Fig. 5| Adoptive transfer of OT-I cells reveals elevated IFN-I in *Ifnar1*⁻^/^⁻** **hosts. A,** Experimental design. Analysis of splenocytes 12 days post adoptive transfer of naïve OT-I cells. **B,** Sca-1^+^ expression of adoptively transferred IFNAR sufficient OT-I CD8 T cells into H8, *Ifnar1*⁻^/^⁻ H8, MHCI.GP, and *Ifnar1*⁻^/^⁻ MHCI.GP hosts. Data show 2-4 biological replicates per group from one experiment. Data points indicate individual mice, with lines indicating data median. P values were calculated with ANOVA with Tukey’s post hoc test. ns=not significant, *p<0.05, ****p<0.0001.

**Supp. Fig. 6| A, General gating strategy** to determine P14 cell **B,** phenotype and **C,** function.
