# Supplementary material for: CD8 T Cell Sensing of Type I Interferon Impacts Anergy
Source: Eur J Immunol. 2026 May 7;56:e70192. doi: 10.1002/eji.70192 (PMC13150955; doi:10.1002/eji.70192)

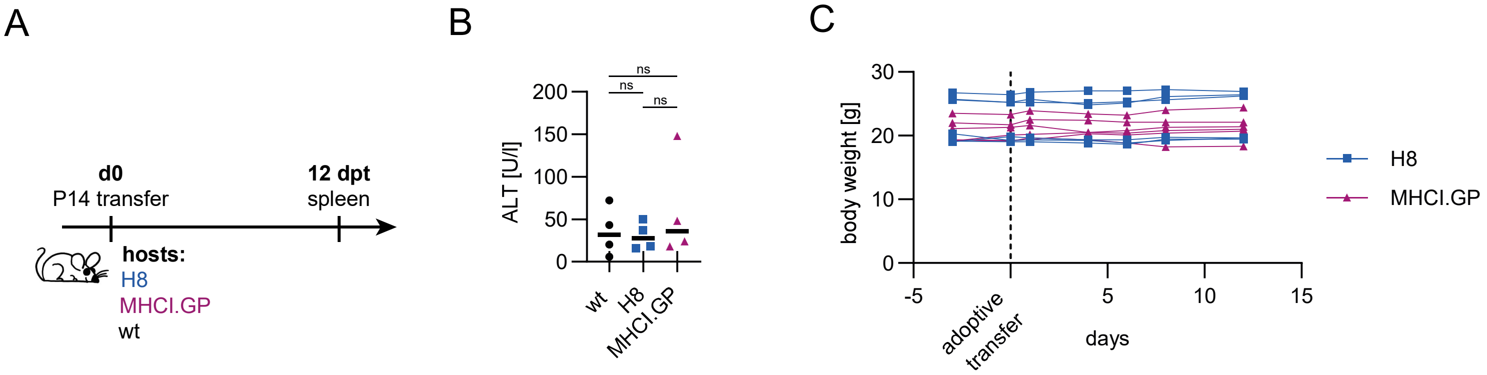
A
d0
P14 transfer
12 dpt
spleen
hosts:
H8
MHCI.GP
wt
B
ns
ns
ns
ALT [U/l]
200
150
100
50
0
wt
H8
MHCI.GP
C
body weight [g]
30
20
10
0
-5
5
10
15
adoptive transfer
days
H8
MHCI.GP

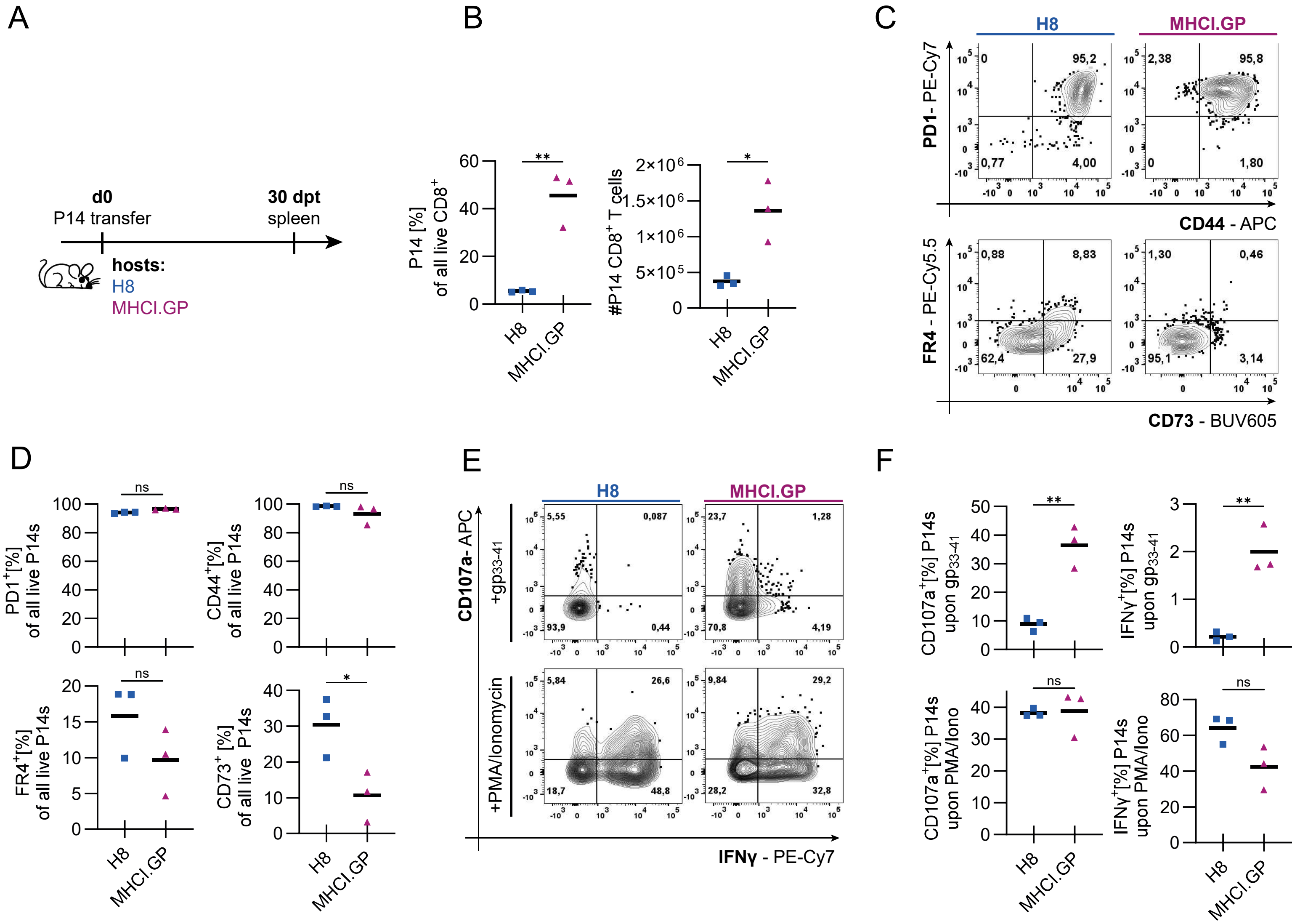
A
d0
P14 transfer
30 dpt
spleen
hosts:
H8
MHCI.GP
B
P14 [%] of all live CD8+
#P14 CD8+ T cells
H8
MHCI.GP
C
H8
MHCI.GP
PD1- PE-Cy7
CD44 - APC
FR4 - PE-Cy5.5
CD73 - BUV605
D
PD1+[%] of all live P14s
CD44+[%] of all live P14s
FR4+[%] of all live P14s
CD73+ [%] of all live P14s
E
CD107a- APC
+gp33-41
+PMA/Ionomycin
IFNγ - PE-Cy7
F
CD107a+[%] P14s upon gp33-41
IFNγ+[%] P14s upon gp33-41
CD107a+[%] P14s upon PMA/Iono
IFNγ+[%] P14s upon PMA/Iono

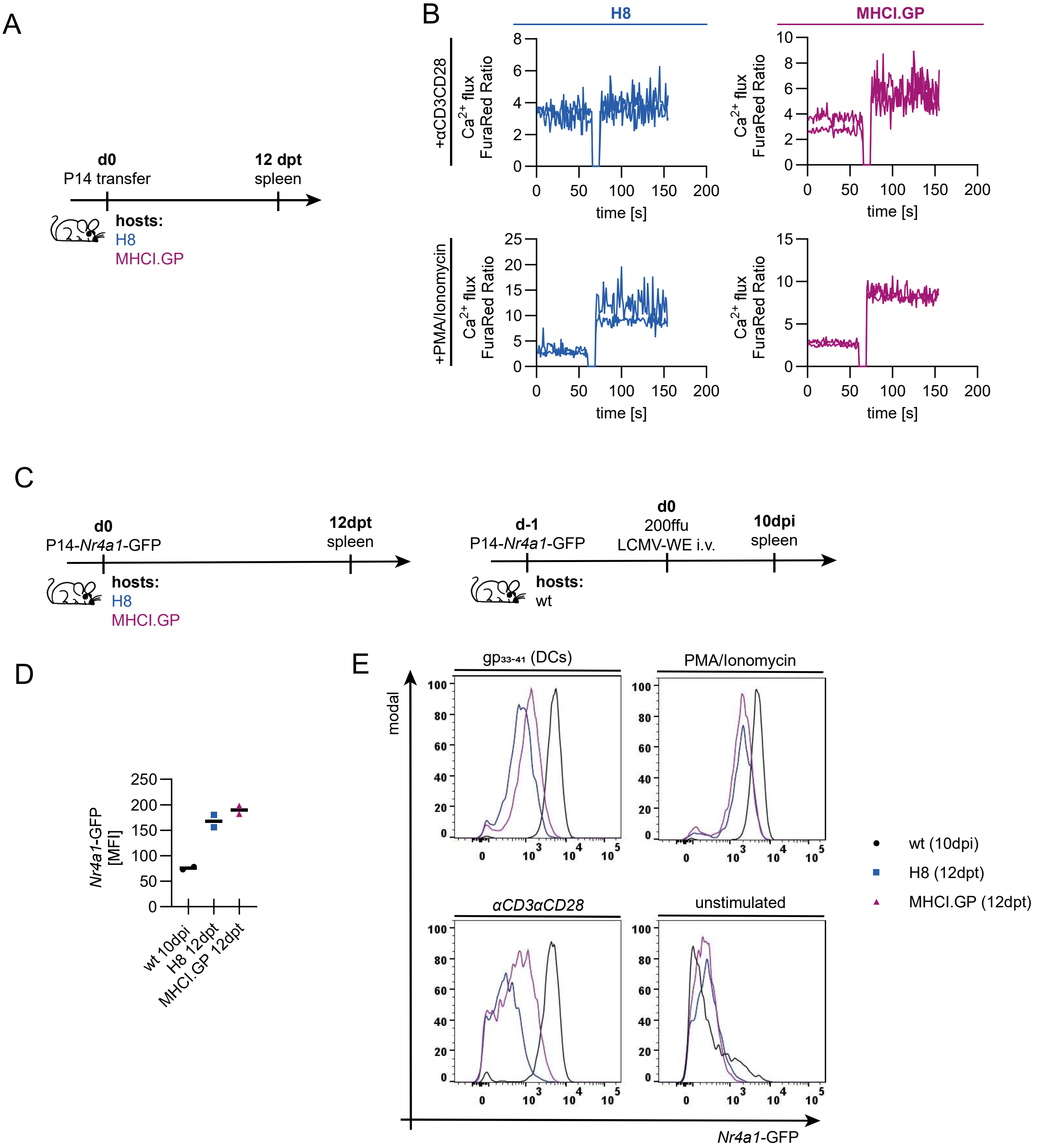
A
d0
P14 transfer
12 dpt
spleen
hosts:
H8
MHCI.GP
B
H8
MHCI.GP
+αCD3CD28
+PMA/Ionomycin
Ca2+ flux FuraRed Ratio
time [s]
C
d0
P14-Nr4a1-GFP
12dpt
spleen
hosts:
H8
MHCI.GP
d-1
P14-Nr4a1-GFP
d0
200ffu
LCMV-WE i.v.
10dpi
spleen
hosts:
wt
D
Nr4a1-GFP [MFI]
wt 10dpi
H8 12dpt
MHCI.GP 12dpt
E
gp33-41 (DCs)
PMA/Ionomycin
αCD3αCD28
unstimulated
modal
Nr4a1-GFP
wt (10dpi)
H8 (12dpt)
MHCI.GP (12dpt)

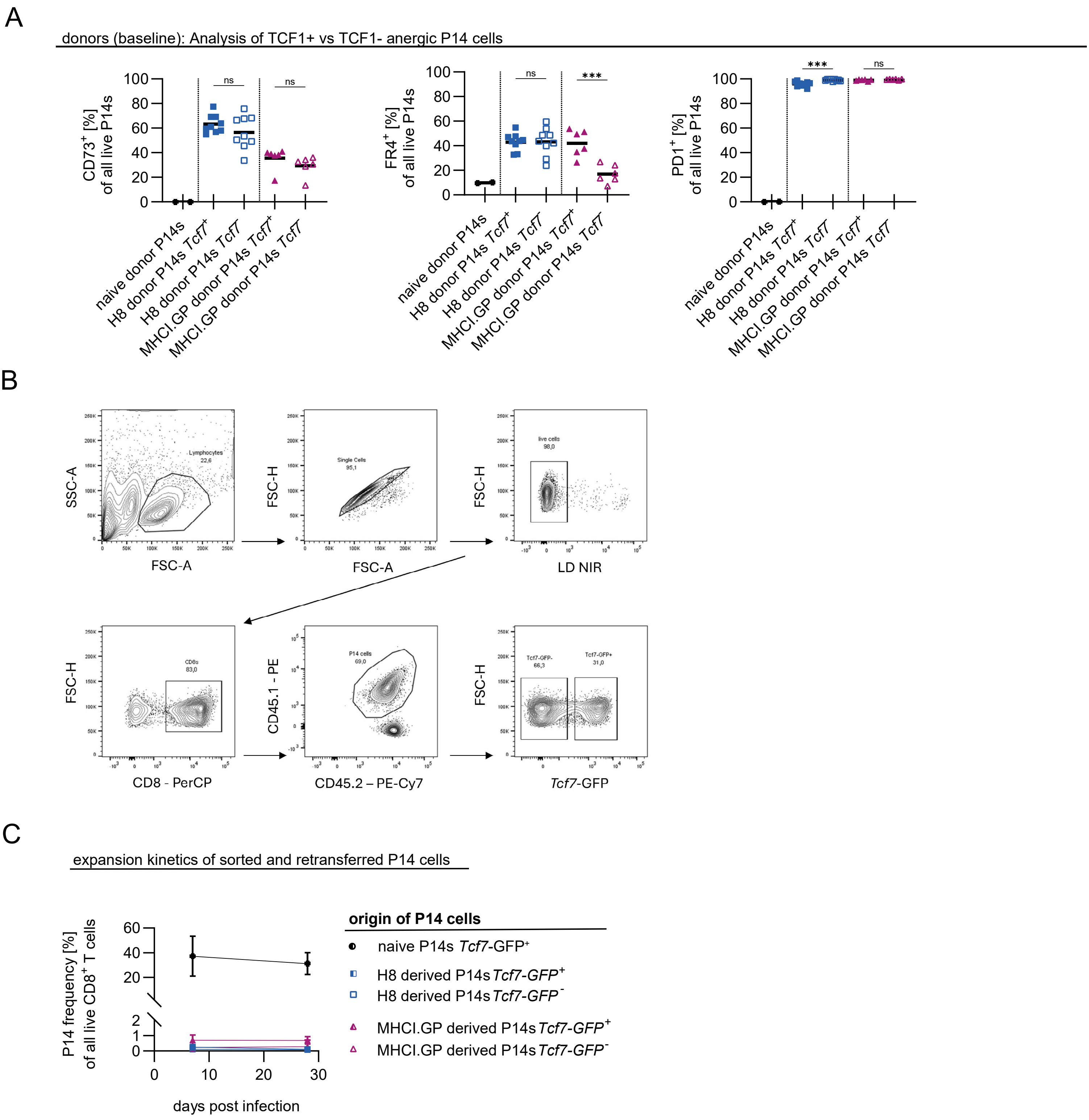
A
donors (baseline): Analysis of TCF1+ vs TCF1- anergic P14 cells
CD73+ [%] of all live P14s
FR4+ [%] of all live P14s
PD1+ [%] of all live P14s
ns
***
naive donor P14s
H8 donor P14s Tcf7+
H8 donor P14s Tcf7-
MHCI.GP donor P14s Tcf7+
MHCI.GP donor P14s Tcf7-
B
SSC-A
FSC-A
Lymphocytes
22,6
FSC-H
Single Cells
95,1
live cells
98,0
LD NIR
CD8s
83,0
CD8 - PerCP
CD45.1 - PE
P14 cells
69,0
CD45.2 – PE-Cy7
Tcf7-GFP-
66,3
Tcf7-GFP+
31,0
Tcf7-GFP
C
expansion kinetics of sorted and retransferred P14 cells
P14 frequency [%] of all live CD8+ T cells
days post infection
origin of P14 cells
naive P14s Tcf7-GFP+
H8 derived P14s Tcf7-GFP+
H8 derived P14s Tcf7-GFP-
MHCI.GP derived P14s Tcf7-GFP+
MHCI.GP derived P14s Tcf7-GFP-

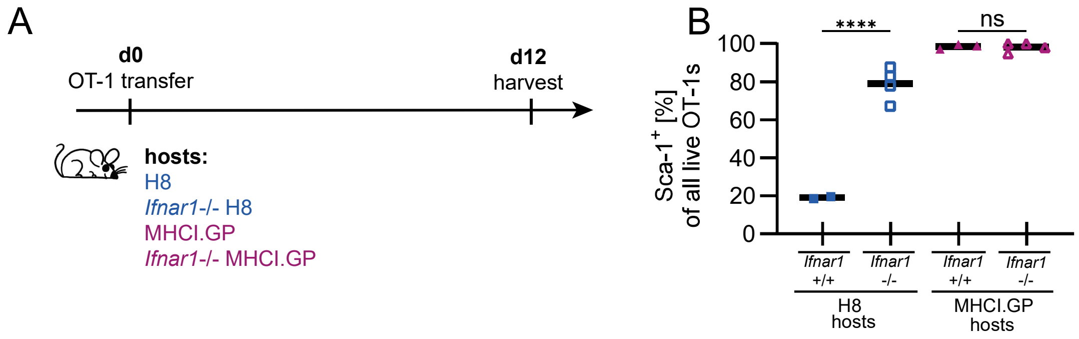
A
d0
OT-1 transfer
d12
harvest
hosts:
H8
Ifnar1-/- H8
MHCI.GP
Ifnar1-/- MHCI.GP
B
Sca-1+ [%] of all live OT-1s
100
80
60
40
20
0
****
ns
Ifnar1 +/+
Ifnar1 -/-
Ifnar1 +/+
Ifnar1 -/-
H8 hosts
MHCI.GP hosts

A

Standard gating strategy for P14 cells:

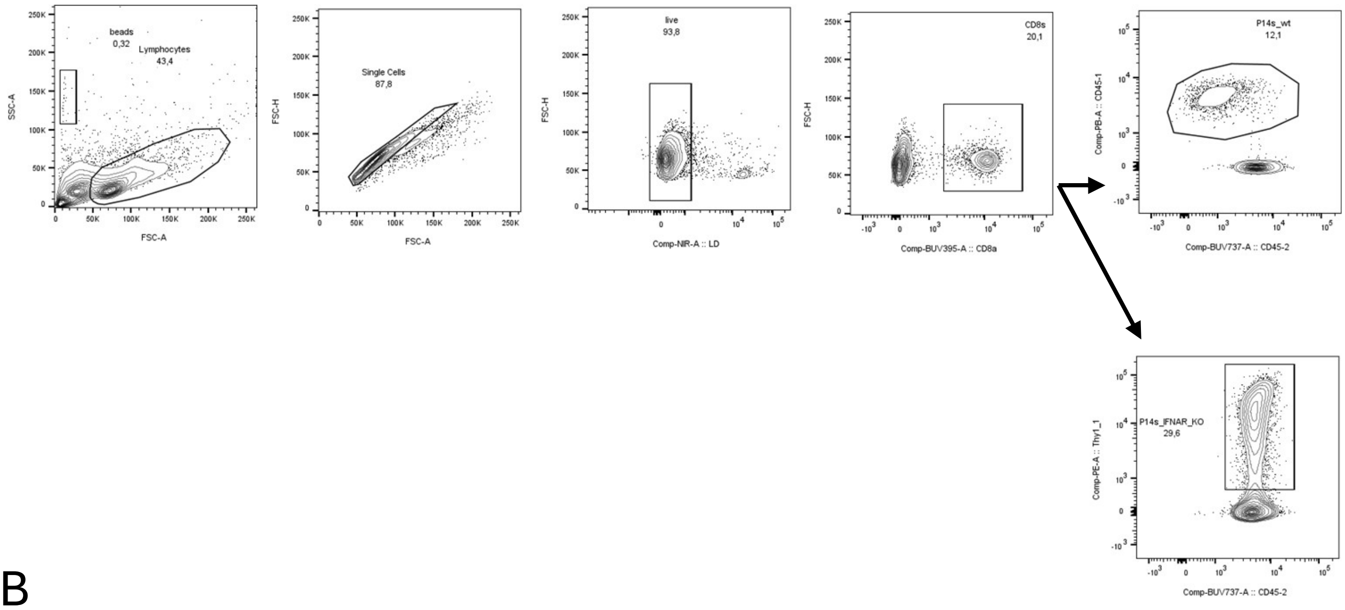

B

Plots gated on single, live, CD8+, CD45.1+/Thy1.1+ P14 cells:

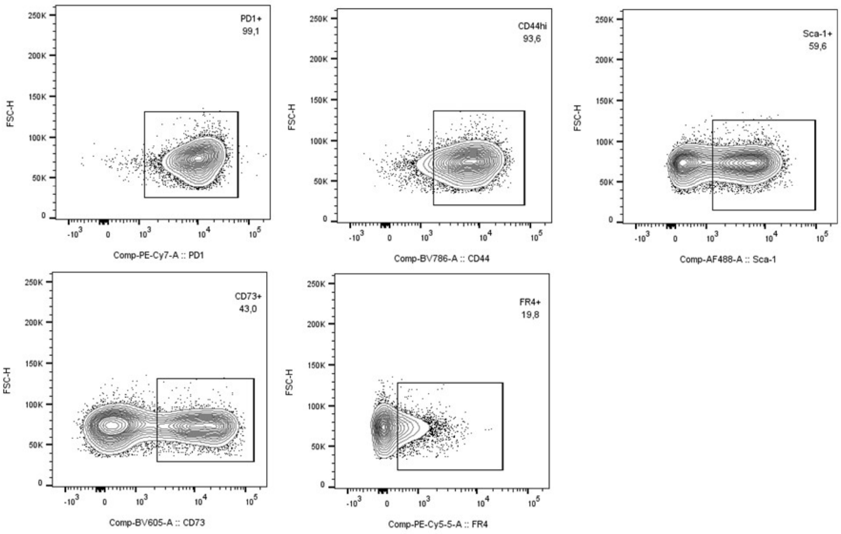

C

Plots gated on single, live, CD8+, CD45.1+/Thy1.1+ P14 cells:

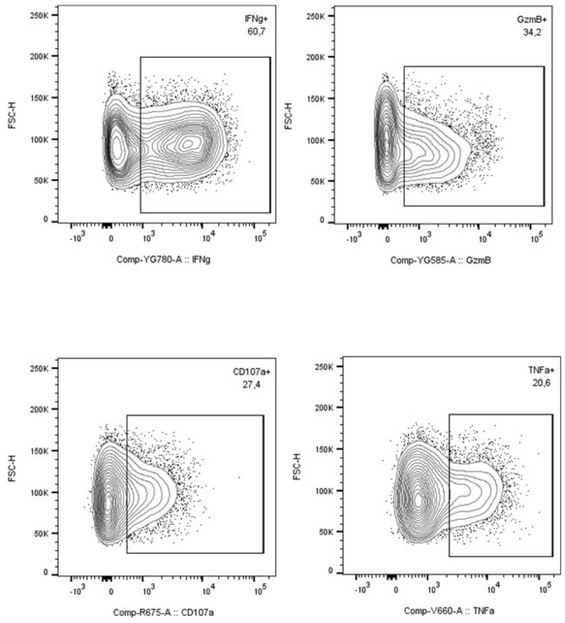

Supplement: Supplementary file 2 — Supporting File 2: eji70192‐sup‐0002‐Figures.pdf. [file EJI-56-e70192-s001.pdf]
